# Evaluation of post-operative complications after mastectomy performed without perioperative antimicrobial prophylaxis in dogs

**DOI:** 10.1186/s13028-021-00600-3

**Published:** 2021-08-30

**Authors:** Philip Spåre, Ingrid Ljungvall, Karl Ljungvall, Annika Bergström

**Affiliations:** 1 IVC (Independent Vetcare) Evidensia South Animal Hospital, Månskärsvägen 13, 141 75 Kungens Kurva, Sweden; 2 Department of Clinical Sciences, Swedish University of Agricultural Sciences, Box 7054, 75007 Uppsala, Sweden; 3 AniCura Animal Hospital, Rinkebyvägen 21A, 18236 Danderyd, Sweden

**Keywords:** Antibiotics, Complication, Mammary tumours, Mastectomy, Surgical site infection

## Abstract

**Background:**

Mastectomy is the most common procedure for treatment of mammary tumours. Dogs undergoing mastectomy have a risk of developing surgical site infections (SSI) and other postoperative complications. However, potential risk factors associated with such complications have been sparsely investigated. Thus, the objective of this retrospective study was to determine the incidence of, and identify risk factors for, SSI and non-SSI postoperative complications after mastectomy performed without perioperative antimicrobial prophylaxis in privately owned otherwise clinically healthy dogs.

**Results:**

Medical records were reviewed retrospectively for 135 client-owned female dogs, 10–35 kg in weight and three to 10 years of age, which had undergone mastectomy due to mammary tumours at three referral animal hospitals in Sweden over a 3-year period. Twelve (8.9%) dogs developed SSI, and 21 dogs (17.1%) dogs suffered a non-SSI postoperative complication. The incidence of SSI and all complications (SSI and non-SSI) were higher in dogs that had two to three (SSI: P = 0.036 and all complications: P = 0.0039) and four to five (SSI and all complications: P = 0.038) mammary glands excised, compared to dogs that had one mammary gland excised. The incidence of SSI was 1.7% (n = 1/60) in dogs that had one gland removed. The incidence of non-SSI postoperative complications was higher in dogs with a higher body weight (P = 0.02).

**Conclusions:**

The incidence of SSI was lower than or similar to previously reported incidences of SSI in dog populations that have undergone tumour excisional surgery, despite the fact that dogs in the present study had not received perioperative antibiotics. Dogs that had two or more glands excised had an increased risk of developing SSI and non-SSI complications compared to dogs that had one gland excised. Furthermore, higher BW was associated with an increased risk of non-SSI complications. Results from the study indicate that routine use of perioperative antibiotics in tumour excisional surgery can be questioned, at least in single gland mastectomy in otherwise clinically healthy dogs.

## Background

Mammary tumours are one of the most common types of neoplasia in female dogs and account for about half of all tumours in intact female dogs [1–4]. Mastectomy is the most common procedure for treatment of mammary tumours [5]. Dogs undergoing mastectomy, as well as dogs undergoing other types of skin and reconstructive surgery, have a risk of developing surgical site infection (SSI) and other postoperative complications [6–8]. To minimise the risk of SSI, the surgeon should take several pre-, intra- and postoperative precautions. Pre- and intraoperative precautions include stringent cleaning of the surgical area and aseptic surgical techniques, minimisation of dead space, efficient haemostasis and atraumatic tissue handling, including avoidance of excessive stretching of the skin when closing the wound [9–12].

Despite meticulous technique and careful precautions, SSI can occur and remains an important cause of postoperative morbidity [13]. Perioperative antimicrobial therapy has been suggested to prevent SSI in dogs undergoing reconstructive surgery [6, 8], and perioperative antimicrobial therapy has been recommended by some authors for procedures where surgical time exceeds 90 min [13]. However, because multi-resistant bacteria have emerged as a world-wide health problem, various factors associated with the development of SSI and other postoperative complications need to be further elucidated in patients undergoing surgery in order to potentially reduce routine use of antimicrobial perioperative prophylaxis.

Thus, the objective of this retrospective study was to determine the incidence of, and identify risk factors for, SSI and non-SSI postoperative complications after mastectomy performed without perioperative antimicrobial prophylaxis in privately owned otherwise clinically healthy dogs.

## Methods

### Dog population

Medical records were reviewed retrospectively from three referral animal hospitals in Sweden: the University Animal Hospital at the Swedish University of Agricultural Sciences in Uppsala, Evidensia South Animal Hospital in Stockholm, and AniCura Albano Animal Hospital in Stockholm. Records from female dogs that had undergone standardised surgical procedures to remove mammary tumours, ranging from single gland mastectomies to unilateral total mastectomies, between 1 July 2013 and 30 September 2016 were evaluated. Dogs that had undergone previous mastectomy within a 2-month period prior to the surgery were excluded. To avoid certain individuals being given too much weight in the analysis, only the first surgery for each dog during the time period was included. Dogs of any breed were eligible for inclusion in the study if they were between the ages of three to 10 years at the time of surgery and had a body weight (BW) between 10 and 35 kg. Only dogs with information available in the medical records regarding the wound healing process during the first month postoperatively were included.

Dogs suffering significant concurrent systemic or organ related diseases or disorders that could affect wound healing (e.g. endocrine and skin disease processes) were excluded. Dogs were also excluded if they had been treated with perioperative antibiotics during the mastectomy or if they had been treated with substances at the time of surgery that potentially could affect wound healing, such as corticosteroids. Furthermore, dogs that had undergone surgery where several non-coherent glands had been excised, where solely lumpectomies had been performed, or concurrent surgery not concerning excision of mammary gland tumours (e.g. celiotomies or excision of non-mammary gland tumours) were excluded from the study.

### Medical record information

All medical records from dogs included in the study were studied at a minimum of 1 month postoperatively. Patient records and bacteriological culture results were compiled with owner permission.

All records were compiled by the same veterinarian (PS). The following variables were registered in the dataset: dog breed; age; BW; neutering status; season (defined as winter: December until the end of February; spring: March until the end of May; summer: June until the end of August; and fall: September until the end of November) when surgery was performed, hospital (1–3); number of mammary glands excised (group 1—one gland excised; group 2—two or three glands excised; or group 3—four or five glands excised); anatomical localisation of glands excised (cranial glands, defined as number one to three; caudal glands, defined as number four to five; or a combination of both); length of anaesthesia (defined from the time of induction to the time of extubation); type of tumour/s excised; SSI or non-SSI postoperative complication, of which the latter included, but was not limited to, seroma, dehiscence and suture reaction; number of days postoperatively when complications occurred; and results from potential bacterial culture. Dogs that developed an SSI could not also be registered as having a non-SSI postoperative complication, because such complications could be associated with the development of SSI, and the cause-effect relationship might therefore be unclear. Verification of a diagnosis of SSI in a dog required that signs described in the medical records fulfilled the necessary criteria (Table 1), which were adapted and modified from the Centers for Disease Control and Prevention (CDC) guidelines [14]. A dog that did not fulfil the necessary criteria for SSI according to CDC-guidelines, and that had a positive bacterial growth cultured from the incision considered as normal skin microbiota, was not considered to suffer from SSI. All suspected SSI cases were evaluated and verified based on the information gained from the journals by two of the authors (PS and AB).

**Table. 1:** Criteria for diagnosis of superficial incisional surgical site infection

Criteria for diagnosis of superficial incisional SSI:
Date of event occurred within 30 days after surgical procedure, where day 1 was the procedure date
AND Only the skin and the subcutaneous tissue of the incision were involved
AND The patient had at least one of the following signs of infection:
• Purulent drainage from the superficial incision
• Organism(s) was (were) identified from an aseptically-obtained specimen from the superficial incision or subcutaneous tissue by a culture or non-culture based microbiologic testing method, which was performed for purposes of clinical diagnosis or treatment
• Superficial incision had been deliberately opened by a surgeon but culture or non-culture based testing of the superficial incision or subcutaneous tissue had not been performed
AND Patient had at least one of the following clinical signs:
Localized pain or tenderness; localized swelling; erythema; and/or heat

The criteria used were adapted and modified after Centers for Disease Control and Prevention (CDC) guidelines [14]. The criteria described had to be met for a dog to be given a diagnosis of superficial incisional surgical site infection (SSI) in the study

Tumours were classified as malignant or benign based on the pathology report.

### Statistical analyses

Statistical analysis was performed using commercially available software.^1^ Data are presented as medians and interquartile range (IQR). A value of P < 0.05 was considered significant for all analyses. Univariable logistic regression analysis was used to analyse potential associations between SSI and non-SSI postoperative complications, individually and in combination, and season, hospital, age, BW, neuter status, number and anatomical localisation of glands excised, length of anaesthesia, and type of tumour/s excised. Odds ratio and 95% confidence intervals were calculated.

## Results

Data from a total of 135 female dogs were included in the study (Table 2). These consisted of 23 mixed breed dogs, nine English Springer Spaniels, nine Labrador Retrievers, seven Cocker Spaniels and 51 different other breeds with one to five dogs represented from each breed. Fifty-five, 43 and 37 dogs, respectively, were recruited from the three hospitals. Forty-two surgeries were performed in the summer, 37 in the autumn, 31 in the winter and 25 in the spring.

**Table. 2:** Age, bodyweight, neutering status, number of mammary glands removed and incidence of postoperative complications; including surgical site infection (SSI), in 135 female dogs with mammary tumours

Dog characteristics (n = 135)			Mammary glands removed 1/2–3/4–5	Postoperative complications	
Age (years)	BW (kg)	Intact/neutered			
8.2 (7–9.4)	21.6 (15.5–27.2)	111/24	60/57/18	SSI yes/no	12/135
				Other complications yes/no^a^ Seroma Dehiscence Reaction towards the suture material Other	21/135
					n = 9 n = 4 n = 1 n = 7

^a^Other complications include signs of mild redness or swelling without exudation or pain

In all of the surgeries, anaesthesia was induced with propofol and maintained with inhalation anaesthesia, using isoflurane or sevoflurane in oxygen. Information about the length of anaesthesia was available for 124/135 (91.9%) of the dogs. The median length of anaesthesia was reported to be 90 min (IQR 65–120) in these 124 dogs, and 63 of the dogs were anesthetized ≤ 90 min and 61 of the dogs were anesthetized > 90 min. The training level of the surgeons ranged from non-specialists to board certified specialists in surgery. Surgeries in all the included dogs was performed according to a standardised procedure: an elliptical incision was made around the mammary gland(s) to be excised. The tissue was subcutaneously dissected with sharp and blunt dissection. If necessary, the fascia was partially removed in case of minimal margins or adherence to the tumour/tumours. Suturing was performed with monofilament sutures in all layers, absorbable in fascia and subcutaneously and non-absorbable in the skin.

**Fig. 1:**
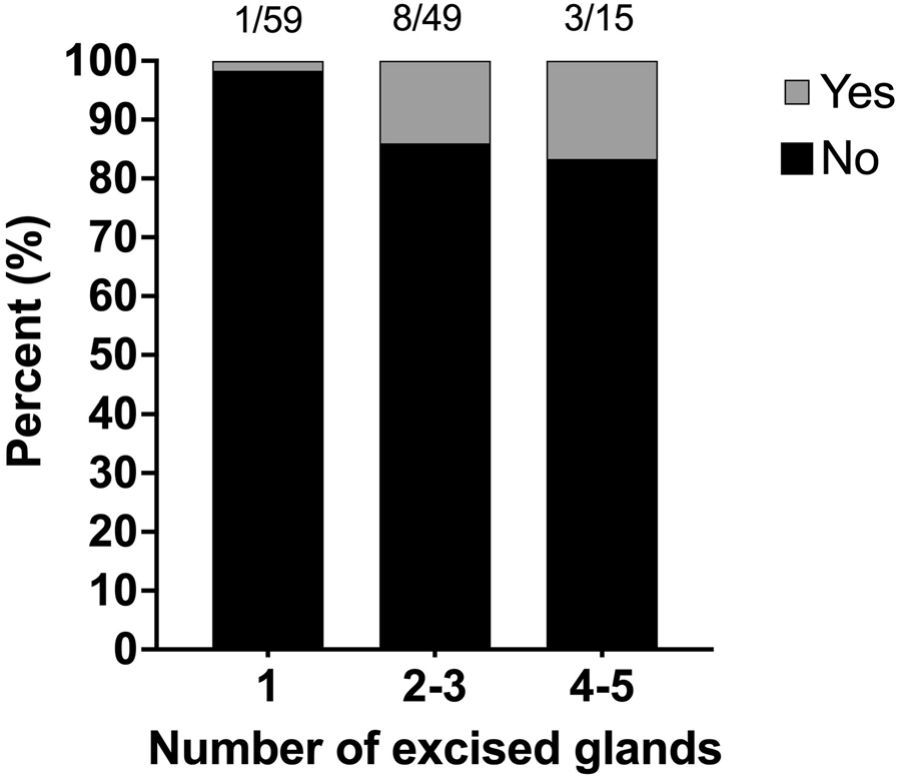
Incidence of dogs that developed surgical site infection postoperatively grouped according to number of glands surgically removed

The number of dogs developing surgical site infection (SSI) and various other postoperative complications is presented in Table 2. The SSIs were detected at a median of 8 days postoperatively (IQR 5–10). The incidence of SSI was 1.7% (n = 1/60) in the dogs that had one gland excised, 14% (n = 8/57) in the dogs that had two to three glands excised, and 16.7% (n = 3/18) in the dogs that had four to five glands excised (Fig. 1).

Bacteriological culture had been obtained in 11 of the 12 dogs deemed affected by SSI, and 10 of these cultures (91%) had positive growth. All cultured bacteria were sensitive to amoxicillin. *Staphylococcus pseudintermedius* was the most common organism cultured (eight cases).

**Fig. 2:**
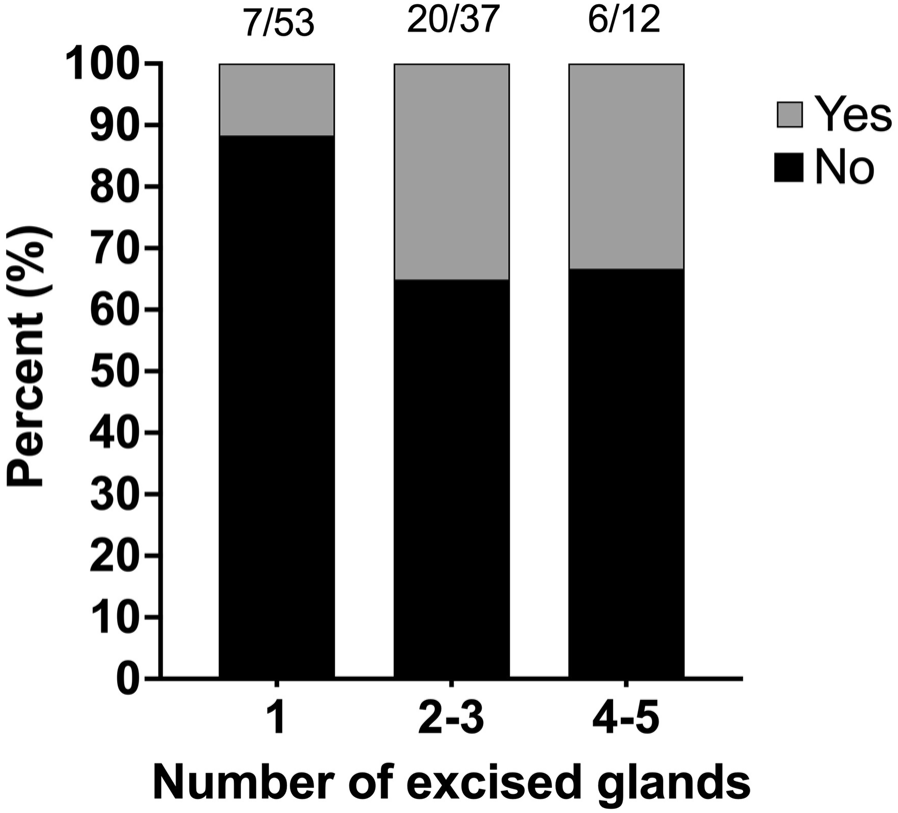
Incidence of dogs that developed any kind of complication postoperatively grouped according to number of glands surgically removed

Non-SSI complications were detected at a median of 9 days postoperatively (IQR 6–13). The incidence of non-SSI postoperative complication was 10.2% (n = 6/59) in the dogs that had one gland excised, 24.5% (n = 12/49) in the dogs that had two to three glands excised, and 20% (n = 3/15) in the dogs that had four to five glands excised (Fig. 2).

A benign tumour was diagnosed by histopathology in 88 dogs (65%) and a malignant tumour in 35 (26%) dogs. The tumour type was determined in 12 (9%) dogs.

### Univariable logistic regression analysis

A positive association was found between diagnosis of SSI and number of mammary glands excised (P = 0.015): the incidence of SSI was higher in dogs that had two to three (OR 9.6, 95% CI 1.2–79.7, P = 0.036) and four to five (OR 11.8, 95% CI 1.1–121.7, P = 0.038) mammary glands excised, compared to dogs that had one mammary gland excised. A positive association was also found between all complications (SSI and non-SSI postoperative complications) and number of mammary glands excised (P = 0.0063): the incidence of all complications was higher in dogs that had two to three (OR 4.1, 95% CI 1.6–10.7, P = 0.0039) and four to five (OR 3.8, 95% CI 1.1–13.3, P = 0.038) mammary glands excised, compared to dogs that had one mammary gland excised. The incidence of non-SSI postoperative complication was higher in dogs with a higher BW (OR 1.1, 95% CI 1.0–1.18, P = 0.020).

## Discussion

In the present study, the incidence of SSI was lower than, or similar to, previously reported incidences in dog populations that have undergone tumour excisional surgery, despite the fact that all mastectomies had been performed without perioperative antimicrobial prophylaxis. Dogs that had undergone more extensive mastectomy were found to have an increased risk of developing post-operative complications compared to dogs that had one gland excised.

The overall incidence of SSI was 8.9% in the present study, which included dogs that had undergone mastectomies ranging from single gland mastectomies to radical mastectomies. The incidence of SSI has previously been reported to be 17% in dogs that have undergone regional mastectomy, 23% in dogs that have undergone radical mastectomy [7], and 9% in dogs that have undergone mastectomies ranging from small lumpecomties to radical mastectomies [15]. All dogs included in these previous published studies had been administered perioperative antibiotics. In contrast, dogs included in the present study had not been treated with perioperative antibiotics. The incidence of SSI of 1.7% in dogs that had one gland excised in the present study population clearly indicates that perioperative antibiotics are superfluous for single gland mastectomy in an otherwise clinically healthy dog. This is in agreement with previously published recommendations [13] and reinforces the indication that the prophylactic use of antibiotics should be based on individual risk assessments. Also, for more extensive mastectomies, the incidence of SSI in our study population was lower than previously reported by Horta et al. [7]. Based on the results from the present study, which are in line with previously published studies [16, 17], the efficacy of the routine prophylactic use of perioperative antibiotics in dogs undergoing mastectomies can be called into question.

Dogs that had two or more glands excised had a higher risk of developing SSI and non-SSI complications compared to dogs that had one gland excised. Dogs that have several glands excised may have had longer anaesthesia and surgery periods, and the length of anaesthesia and surgery has previously been reported to be a risk factor for the development of SSIs [18]. However, the higher incidence of SSI could not be explained by the length of anaesthesia in the present study, which is in agreement with findings from a recently published canine mastectomy study [15]. Dead space has been suggested to be a risk factor for the development of seromas, which, according to human mastectomy studies, may increase the risk of SSI and other morbidities [19–22]. With an increasing number of glands excised, the wound becomes increasingly larger, and as more tissue is removed, the potential dead space increases despite attempts to close dead space by suturing. The tension in the suture line may, furthermore, be higher in larger wounds, leading to disrupted micro circulation and impaired healing, and thereby prolonged damage to the skin barrier and development of SSI [12].

High BW was associated with a higher risk of complications other than SSI. The influence of incisional size, which commonly increases with an increasing dog size, on the development of post-operative complications could potentially describe the association demonstrated. Incisional length has previously been reported to be a risk factor for SSI in horses [23]. Post-operative complications have also previously been shown to be associated with higher BW in dogs [15, 24]. A potential influence of body fat reserves could not be investigated in the current study as information about body condition score was missing in many of the medical records.

Previous studies in humans have indicated positive associations between SSI and warmer periods of the year [25, 26]. Interestingly, we did not see any effect of season on the incidence of SSI.

Study limitations for present study include the retrospective design. The direct consequences of the retrospective study design are that the authors have not examined all the dogs themselves. Additionally, as dogs were excluded if the medical records were not complete with regard to data pertaining to wound healing, a number of dogs without complications may not have been included, as owners or referring clinicians may have removed sutures and provided post-operative care. This may result in an overestimation of the true incidence of post-operative complications based on the current material. Furthermore, conclusions drawn from the present study may not be valid for dogs with concurrent systemic disease processes and dogs older than 10 years, as such dogs were not included in the dog population investigated.

## Conclusions

The incidence of SSI was lower than or similar to previously reported incidences of SSI in dog populations that have undergone tumour excisional surgery, even though dogs in the present study had not received perioperative antibiotics. Dogs that had two or more glands excised had an increased risk of developing SSI and non-SSI complications, compared to dogs that had one gland excised; however, the finding could not be explained by the length of anaesthesia in this retrospective study. Furthermore, higher BW was associated with an increased risk of developing non-SSI complications. Results from the study indicate that the efficacy of the routine use of perioperative antibiotics in tumour excisional surgery, such as mastectomies, can be called into question and may be considered unnecessary, at least in cases where single gland mastectomy is performed in otherwise healthy dogs.

## Data Availability

The datasets analysed during the current study are not publicly available due to regulations under the General Data Protection Regulation, but parts of the data may be available from the corresponding author upon reasonable request.

## Footnotes

1 JMP® Pro version 14.0.0, SAS Institute Inc. Cary, NC.

## References

[1] Egenvall A, Bonnett BN, Ohagen P, Olson P, Hedhammar A, von Euler H (2005). Incidence of and survival after mammary tumors in a population of over 80,000 insured female dogs in Sweden from 1995 to 2002. Prev Vet Med.

[2] Moe L (2001). Population-based incidence of mammary tumours in some dog breeds. J Reprod Fertil Suppl.

[3] Moulton JE, Taylor DO, Dorn CR, Andersen AC (1970). Canine mammary tumors. Pathol Vet.

[4] Gilbertson SR, Kurzman ID, Zachrau RE, Hurvitz AI, Black MM (1983). Canine mammary epithelial neoplasms: biologic implications of morphologic characteristics assessed in 232 dogs. Vet Pathol.

[5] Novosad CA (2003). Principles of treatment for mammary gland tumors. Clin Tech Small Anim Pract.

[6] Field EJ, Kelly G, Pleuvry D, Demetriou J, Baines SJ (2015). Indications, outcome and complications with axial pattern skin flaps in dogs and cats: 73 cases. J Small Anim Pract.

[7] Horta RS, Figueiredo MS, Lavalle GE, Costa MP, Cunha RM, Araujo RB (2015). Surgical stress and postoperative complications related to regional and radical mastectomy in dogs. Acta Vet Scand.

[8] Montinaro V, Massari F, Vezzoni L, Liptak JM, Straw RC, Allen L (2015). Lateral caudal axial pattern flap in 13 dogs. Vet Surg.

[9] Amsellem P. Complications of reconstructive surgery in companion animals. Vet Clin North Am Small Anim Pract. 2011;41:995–1006, vii. 10.1016/j.cvsm.2011.05.005 21889697

[10] Lascelles BD, Thomson MJ, Dernell WS, Straw RC, Lafferty M, Withrow SJ (2003). Combined dorsolateral and intraoral approach for the resection of tumors of the maxilla in the dog. J Am Anim Hosp Assoc.

[11] McDonnell G, Russell AD (1999). Antiseptics and disinfectants: activity, action, and resistance. Clin Microbiol Rev.

[12] Pavletic MM, Pavletic MM (2018). Tension-relieving techniques. Atlas of small animal wound management and reconstructive surgery.

[13] Nelson LL. Surgical site infections in small animal surgery. Vet Clin North Am Small Anim Pract. 2011;41:1041–56, viii. 10.1016/j.cvsm.2011.05.010 21889700

[14] Berrios-Torres SI, Umscheid CA, Bratzler DW, Leas B, Stone EC, Kelz RR (2017). Centers for disease control and prevention guideline for the prevention of surgical site infection, 2017. JAMA Surg.

[15] Evans BJ, Holt DE, Stefanovski D, Sorenmo KU (2021). Factors influencing complications following mastectomy procedures in dogs with mammary gland tumors: 140 cases (2009–2015). J Am Vet Med Assoc.

[16] Dyall B, Schmokel H (2017). Tibial tuberosity advancement in small-breed dogs using TTA rapid implants: complications and outcome. J Small Anim Pract.

[17] Dyall BAR, Schmokel HG (2018). Surgical site infection rate after hemilaminectomy and laminectomy in dogs without perioperative antibiotic therapy. Vet Comp Orthop Traumatol.

[18] Vasseur PB, Levy J, Dowd E, Eliot J (1988). Surgical wound infection rates in dogs and cats. Data from a teaching hospital. Vet Surg.

[19] Aravind B, Cook A (2015). Intra-abdominal giant infected seroma following laparoscopic inguinal hernia repair. Hernia.

[20] Chilson TR, Chan FD, Lonser RR, Wu TM, Aitken DR (1992). Seroma prevention after modified radical mastectomy. Am Surg.

[21] Coveney EC, O'Dwyer PJ, Geraghty JG, O'Higgins NJ (1993). Effect of closing dead space on seroma formation after mastectomy—a prospective randomized clinical trial. Eur J Surg Oncol.

[22] Kuroi K, Shimozuma K, Taguchi T, Imai H, Yamashiro H, Ohsumi S (2006). Effect of mechanical closure of dead space on seroma formation after breast surgery. Breast Cancer.

[23] Darnaud SJ, Southwood LL, Aceto HW, Stefanovski D, Tomassone L, Zarucco L (2016). Are horse age and incision length associated with surgical site infection following equine colic surgery?. Vet J.

[24] Muraro L, White RS (2014). Complications of ovariohysterectomy procedures performed in 1880 dogs. Tierarztl Prax Ausg K Kleintiere Heimtiere.

[25] Kane P, Chen C, Post Z, Radcliff K, Orozco F, Ong A (2014). Seasonality of infection rates after total joint arthroplasty. Orthopedics.

[26] Durkin MJ, Dicks KV, Baker AW, Lewis SS, Moehring RW, Chen LF (2015). Seasonal variation of common surgical site infections: does season matter?. Infect Control Hosp Epidemiol.

